# Comparative analysis of indwelling time and complications of mid-line catheters in different punctured veins of neonates

**DOI:** 10.3389/fped.2025.1577791

**Published:** 2025-07-04

**Authors:** Lijuan Yang, Yixin Wang, Song Luo, Yanju Tao, Xinxin Shi, Wenda Xu, Yanling Li, Caixia Sun, Tingting Lei, Bing Xu

**Affiliations:** ^1^ Intravenous Infusion Group, The First Affiliated Hospital of Bengbu Medical University, Bengbu, China; ^2^ Department of Pediatrics, The First Affiliated Hospital of Bengbu Medical University, Bengbu, China; ^3^ Department of Neurology, The First Affiliated Hospital of Bengbu Medical University, Bengbu, China

**Keywords:** mid-line catheter, complications, neonates, indwelling time, nursing

## Abstract

**Objective:**

We aims to evaluate the effects and complications associated with mid-line catheters inserted via different puncture veins in neonates, ultimately providing a foundation for selecting the most suitable puncture site for catheterization in clinical neonatal practice.

**Methods:**

A retrospective data analysis was conducted, involving 244 neonates with indwelling mid-line catheters who were admitted to a Class III Grade A general hospital in Anhui Province between August 2020 and December 2023. The study compared catheter indwelling duration, the incidence of catheter-related complications, and the first puncture success among neonates with catheterization through various veins.

**Results:**

The analysis revealed a statistically significant difference in indwelling duration across different puncture veins (*H* *=* *28.65, P* *<* *0.001*). Specifically, significant differences were observed in the indwelling duration between the median cubital vein, axillary vein, and superficial temporal vein (adj. *P* < 0.05), whereas no significant differences were found among the other puncture sites (adj. *P* > 0.05). A statistically significant variation in catheter complications was observed among different puncture veins (*P* < 0.001). Specifically, the incidence of complications was lower in the median cubital and axillary veins compared to other puncture sites, with these differences reaching statistical significance (*P* < 0.05). Furthermore, the basilic vein exhibited a lower incidence of complications than the cephalic vein, superficial temporal vein, and great saphenous vein, with the difference being statistically significant only when compared to the great saphenous vein (*P* < 0.05). Additionally, no statistically significant difference was found in the success rate of single puncture among the various puncture veins (*P* > 0.05). However, the one-time successful catheter insertion rate was significantly higher for the median cubital and axillary veins compared to other veins (*P* < 0.05), while no statistically significant differences were observed among the remaining veins.

**Conclusion:**

The insertion of mid-line catheters into the axillary vein and the median cubital vein has been shown to extend catheter indwelling time, enhance the ease of catheter insertion, and decrease the incidence of complications. Consequently, it is advisable to prioritize the axillary vein and median cubital vein for the insertion of mid-to-long catheters in neonates.

## 1 Introduction

Neonates in the Intensive Care Unit (ICU) represent a distinct population frequently confronted with challenges arising from various critical and severe medical conditions. These infants require intravenous access for therapeutic interventions and essential intravenous nutritional support to maintain stable vital signs and ensure normal growth and development (1). Consequently, the establishment and maintenance of intravenous access are paramount in neonatal nursing. Peripheral intravenous catheters (PIVCs) and peripherally inserted central catheters (PICCs) are the two most prevalent forms of vascular access utilized in the Neonatal Intensive Care Unit (NICU) (2, 3). In contrast to the limited indwelling duration associated with PIVCs and the elevated risk of bloodstream infections linked to PICCs (4), the Mini-mid-line Catheter (MC) offers an intermediary option for intravenous vascular access between PIVCs and PICCs (5). The MC, also referred to as the midline catheter, is typically inserted using conventional puncture techniques or ultrasound-guided methods, accessing superficial or deep peripheral veins, with the catheter tip positioned at the distal end of the axilla (6). The prevailing expert consensus indicates that the basilic vein is the preferred vessel for venipuncture in inpatients aged over 18 years (7). However, the optimal vein for puncture in neonates remains unclear. Current scholarly efforts are predominantly directed towards investigating the clinical safety of mid-length catheters (8, 9), and there are few studies on the optimal puncture vein for neonatal MC. In view of this, this study analyzed the relevant data of MC indwelling in 244 neonates and compared the clinical effects of MC punctured through different veins, aiming to provide a basis for the selection of veins for neonatal MC catheterization.

## 2 Subjects and methods

### 2.1 Research subjects

This study encompassed all eligible research subjects in a chronological sequence. neonates who underwent MC catheterization in the Neonatal Intensive Care Unit (NICU) of a Class III Grade A general hospital in Anhui Province between August 2020 and December 2023 were retrospectively selected as research participants. A total of 252 neonates satisfied the inclusion criteria. However, 7 cases were discharged from the hospital against medical advice, and 1 case resulted in mortality, rendering complete data on catheter use and clinical outcomes unobtainable; consequently, these cases were excluded from the analysis. Ultimately, the clinical data of 244 neonates were included in the retrospective analysis.

**Inclusion criteria:**

① neonates with MC indwelling.
② Informed consent forms were signed by the family members.
③ Complete medical records were available, including basic demographic information, catheter—related information, treatment and nursing records during hospitalization, etc.

**Exclusion criteria:**

① Patients whose condition deteriorated during hospitalization and required PICC re—insertion.
② Iatrogenic accidental catheter dislodgement.

**Removal criteria:** neonates who were automatically discharged from the hospital or died.

### 2.2 Methods

#### 2.2.1 Catheterization method

The MC catheterization operation was carried out by the nursing staff or specialized nurses in our hospital who had received professional training, possessed the skills and relevant knowledge of neonatal intravenous catheterization, and had rich experience, in accordance with the “Expert Consensus on Clinical Application of Intravenous Mid—line Catheters” (7). Prior to catheterization, the nursing staff performed a thorough physical assessment of the newborn, which included evaluating vital signs, vascular conditions, coagulation function, and the condition of the skin at the intended puncture site. Based on the findings from this assessment, an appropriate vein for puncture was selected. Measurements were taken for the pre-insertion length (the distance from the intended puncture point along the vein to the mid-clavicular line on the same side) and the circumferences of both upper arms (measured at the midpoint of the line connecting the acromion and the olecranon). A 2Fr*10 cm front-end open MC was utilized for the puncture. The procedure adhered strictly to aseptic operational principles, and ultrasound guidance was employed when necessary to enhance the success rate of the puncture.

During the procedure, the infant was positioned supine with the limb on the side of the puncture abducted at an angle between 45° and 90°. The skin was disinfected, focusing on the puncture site, and a sterile drape was applied to ensure maximum aseptic conditions. A 20-gauge cannula puncture sheath was inserted at an angle ranging from 15° to 30°. Upon observing blood return, the insertion angle was decreased, and the sheath was advanced slightly further. Subsequently, one hand stabilized the needle core holder while the outer cannula was further advanced. The needle core was then carefully withdrawn, and a mid-line catheter was introduced through the outer cannula port to a pre-measured length. Blood was aspirated, and a small volume of normal saline was injected to confirm catheter functionality post-insertion. Following this, the cannula puncture sheath was removed, a needle-free connector was attached, and 2 to 3 ml of heparin solution at a concentration of 10 U/ml was utilized for positive-pressure tube sealing. An alginate dressing was applied to the puncture site, and a 6 cm by 7 cm sterile transparent dressing was used to secure the catheter without exerting tension. The disc and extension tube were fixed using the 3 M tape lock + high—lift platform method.

After successful catheter insertion, the parameters such as the catheter model, insertion depth, and arm circumference were accurately recorded, and the catheter was properly fixed. For chest x-rays taken due to the condition during the treatment of this study, the position of the catheter tip was determined according to the catheter tip judgment criteria.

#### 2.2.2 Catheter maintenance method

After catheterization, the mid—line catheter was maintained according to the standardized catheter maintenance protocol established by our hospital. Specifically, it included the following aspects:

– The transparent dressing was changed every 7 days. If the dressing was found to be contaminated, wet, or loose, it was replaced immediately.
– The flushing and locking of the catheter followed the ACL (Assess—Clear—Lock) principle and the SASH steps (10). (S: Flush the catheter with 0.9% normal saline before infusion to assess the catheter function; A: Infuse the medication; S: Flush the catheter with 0.9% normal saline after infusion; H: Use 10 U/ml heparin solution for positive—pressure locking of the catheter after infusion.)
– The puncture vein was closely observed for any abnormal conditions such as redness, swelling, bleeding, or fluid leakage. Meanwhile, the vital signs of the newborn, including body temperature and heart rate, were also monitored. If any abnormalities were found, the doctor was reported immediately and corresponding measures were taken.

### 2.3 Evaluation indicators

#### 2.3.1 Catheter indwelling time

It refers to the time from the successful insertion of the catheter to its removal or the time when the catheter can no longer be used normally due to various reasons (such as catheter blockage, fluid leakage, displacement, etc.). The time is recorded in days.

#### 2.3.2 Incidence of catheter—related complications

According to the Clinical Nursing Practice Guidelines for Common Complications of Venous Catheters (11), catheter-related complications encompass the occurrence of phlebitis, exudation/extravasation, catheter blockage, catheter-related venous thrombosis, and catheter-related bloodstream infections, among others, during both the catheterization process and the indwelling period. The incidence rate is calculated as the proportion of cases exhibiting phlebitis, catheter blockage, bleeding, fluid leakage, or catheter-related bloodstream infections during the catheter's indwelling period. In instances where a single pediatric patient experiences multiple complications concurrently, it is recorded as a single case of complications. The study employed a double-blind design, ensuring that both outcome assessors and data extractors were blinded to the grouping information. The assessment of catheter-related complications was conducted by two nurses specializing in intravenous therapy, who were independent of the study team and not involved in patient grouping or intervention procedures. These nurses remained unaware of the catheter type and study hypothesis throughout the process. The diagnosis of all complications adhered strictly to the definitions outlined in the relevant guidelines.

#### 2.3.3 First puncture success rate

This metric is defined as the ratio of patients, both adult and pediatric, who experience a successful single-needle puncture performed by the clinician, to the total number of patients, both adult and pediatric, undergoing the puncture procedure.

#### 2.3.4 One—time successful catheter insertion rate

It is the proportion of the number of cases where the catheter can be inserted into the blood vessel to the ideal position in one attempt without secondary or multiple adjustments during the puncture process to the total number of punctures.

### 2.4 Data collection

The data of this study were derived from the electronic medical record system of a tertiary first-class general hospital in Anhui Province from August 2020 to December 2023. A research group was established, and two members used a self—designed MC data collection form to gather, organize, and input data from the hospital's Yihui and HIS electronic medical record systems. When there is contradictory data, sough for the this research member to verify it. The collected data included the following four parts:

① General information: It included information such as gender, gestational age, birth weight, and Apgar score at 5 min after birth.
② Catheter insertion information: It included details like the type, model, and manufacturer of the catheter, the punctured vein, the depth of catheter insertion, the date and time of catheter placement, the operator performing the catheterization, whether the first—time puncture was successful, and whether the catheter was inserted smoothly.
③ Catheter maintenance information: It included the date and time of each dressing change, the date and time of heparin cap replacement, the exposed length of the catheter, the arm circumference, and whether there were symptoms such as redness, bleeding, or fluid leakage at the punctured vein.
④ Clinical outcome information collection: It included the final outcome of the catheter (whether it was removed due to complications and the reason for removal was specified), the catheter indwelling time, and the occurrence of catheter—related complications.

### 2.5 Statistical methods

Statistical analysis was performed using SPSS 26.0 software. For measurement data that conformed to a normal distribution, the mean ± standard deviation (±s) was used for statistical description, and the *t*-test was used for comparison between groups. If the data did not conform to a normal distribution, the median (interquartile range, IQR) [M (P25—P75)] was used for statistical description, and non—parametric tests were used for comparison between groups. Count data were expressed as frequencies and percentages, and the chi—square test was used for comparison between groups. The Bonferroni correction was used for pairwise comparisons, if applicable, and a *P* value less than 0.05 was considered to indicate a statistically significant difference.

## 3 Results

### 3.1 Comparison of general data of neonates with different puncture veins

Between August 2020 and December 2023, a total of 252 mid-line catheter insertions were performed at our center. Following the exclusion of seven cases involving neonates who were voluntarily discharged and one case of mortality, data from 244 neonates were included in the final analysis. Among these, catheterizations were performed via the median cubital vein in 116 cases, the basilic vein in 9 cases, the axillary vein in 51 cases, the superficial temporal vein in 51 cases, the cephalic vein in 15 cases, and the great saphenous vein in 2 cases. Prior to catheterization, there were no statistically significant differences in the baseline characteristics of neonates across the different venous access sites (*P* *>* *0.05*), as detailed in Table 1.

**Table 1:** Baseline characteristics of different puncture sites neonates.

Items	Median cubital vein (*n* = 116)	Basilic vein (*n* = 9)	Axillary vein (*n* = 51)	Superficial temporal vein (*n* = 51)	Cephalic vein (*n* = 15)	Great saphenous vein *n* = 2)	Test statistic	*P*
Gestational age (week)	35.03 ± 2.74	35.78 ± 2.91	35.55 ± 3.40	36.33 ± 3.00	35.27 ± 3.28	39.50 ± 0.71	2.12^a^	0.06
Gender [*n* (%)]							2.20^b^	0.85
Male	73 (62.9)	6 (66.7)	31 (60.8)	28 (54.9)	10 (66.7)	2 (100.0)		
Female	43 (37.1)	3 (33.3)	20 (39.2)	23 (45.1)	5 (33.3)	0 (0.0)		
Blood transfusion [*n* (%)]							4.55^b^	0.41
Yes	6 (5.2)	0 (0.0)	1 (2.0)	4 (7.8)	2 (13.3)	0 (0.0)		
No	110 (94.8)	9 (100.0)	50 (98.0)	47 (92.2)	13 (86.7)	2 (100.0)		
Edema [*n* (%)]							1.66^b^	0.91
Yes	9 (7.8)	0 (0.0)	5 (9.8)	6 (11.8)	1 (6.7)	0 (0.0)		
No	107 (92.2)	9 (100.0)	46 (90.2)	45 (88.2)	14 (93.3)	2 (100.0)		
Weight (g)	2251.7 ± 570.1	2211.1 ± 528.9	2412.9 ± 500.8	2509.5 ± 417.6	2315.67 ± 588.97	2600.0 ± 707.1	2.11^a^	0.07
Platelet (10^−9^/L)	247.9 ± 79.6	254.0 ± 157.7	283.02 ± 103.44	283.0 ± 103.4	245.7 ± 69.6	324.0 ± 59.4	1.93^a^	0.09
Albumin (g/L)	34.1 ± 3.2	35.4 ± 4.9	33.8 ± 3.4	34.1 ± 3.6	33.8 ± 3.4	33.7 ± 0.6	0.81^a^	0.54
Hemoglobin (g/L)	171.8 ± 25.5	169.6 ± 24.8	164.9 ± 23.2	163.1 ± 27.2	165.3 ± 23.6	145.5 ± 31.8	1.37^a^	0.24
Hematocrit (L/L)	0.49 ± 0.07	0.47 ± 0.08	0.48 ± 0.06	0.47 ± 0.08	0.49 ± 0.07	0.39 ± 0.06	1.51^a^	0.19

^a^ F value.

^b^ Fisher's exact test.

### 3.2 Comparison of the incidence rates of complications related to different punctured venous catheters

The incidence rates of complications associated with the various puncture sites were as follows: 0% for the axillary vein, 0% for the basilic vein, 8.6% for the median cubital vein, 33.3% for the superficial temporal vein, 46.7% for the cephalic vein, and 100% for the great saphenous vein. These differences were statistically significant (*P* *<* *0.001*). The incidence rates of complications in the median cubital vein and axillary vein were lower than those in other punctured veins, and the differences were statistically significant (*P* *<* *0.05*). The incidence rate of complications in the basilic vein was lower than those in the cephalic vein, superficial temporal vein, and great saphenous vein. The difference was statistically significant when compared with the great saphenous vein (*P* *<* *0.05*), while there was no statistically significant difference when compared with the cephalic vein and superficial temporal vein (*P* *>* *0.05*, Table 2).

**Table 2:** Comparison of catheter-related complications at different puncture sites.

Punctured vein	*n*	Complications
Median cubital vein	116	10 (8.6)^a^
Basilic vein	9	0 (0.0)^a^^,^^b^
Axillary vein	51	0 (0.0)^a^
Superficial temporal vein	51	17 (33.3)^b^^,^^c^
Cephalic vein	15	7 (46.7)^b^^,^^c^
Great saphenous vein	2	2 (100.0)^c^
Fisher's exact test		45.129
*P*		<0.001

^a,b,c^ Respectively represent different subsets of punctured blood vessel categories, there is no statistical difference between the punctured blood vessels with the same letter, and *P* > 0.05.

### 3.3 Comparison of indwelling times of catheters in different punctured veins

The duration of catheter indwelling across various punctured veins was as follows: median cubital vein, 13 days (IQR: 10, 18); basilic vein, 13 days (IQR: 11, 19); cephalic vein, 10 days (IQR: 7, 14); superficial temporal vein, 11 days (IQR: 7, 13); axillary vein, 14 days (IQR: 10, 17); and great saphenous vein, 4 days (IQR: 4, 5.5). The maximum recorded indwelling duration was 42 days, observed in the axillary vein group. Statistically significant differences were identified in catheter indwelling times among the various punctured veins (*H* *=* *28.65, P* *<* *0.001*). Notably, significant differences were observed between the median cubital vein, axillary vein, and superficial temporal vein (*adj.P* *<* *0.05*), whereas no significant differences were found among the other punctured veins (*adj.P* *>* *0.05*, Table 3).

**Table 3:** Comparison of catheter indwelling time at different puncture sites.

Punctured vein	*n*	Indwelling time
Median cubital vein	116	13 (10, 18)
Basilic vein	9	13 (11, 19)
Axillary vein	51	14 (10, 17)
Superficial temporal vein	51	11 (7, 13)^#^^,^^※^
Cephalic vein	15	10 (7, 14)
Great saphenous vein	2	4 (4, 5.5)
Kruskal—Wallis test		28.65
*P*		<0.001

Compared with the median cubital vein, ^#^*P* < 0.05; Compared with the axillary vein, ^※^*P* < 0.05.

### 3.4 Comparison of the first—puncture success rate and the one—time smooth catheter insertion rate among different punctured veins

There was no statistically significant difference in the first—puncture success rate among different punctured veins (*P* *>* *0.05*). The one—time smooth catheter insertion rate of the median cubital vein and the axillary vein was better than that of other punctured veins, and the difference was statistically significant (*P* *<* *0.05*). There was no statistically significant difference in the comparison among the remaining punctured veins (*P* *>* *0.05*, Table 4).

**Table 4:** Comparison of different puncture sites on first puncture success and one-time smooth catheter insertion.

Punctured vein	*n*	First puncture success	One-time smooth catheter insertion
Median cubital vein	116	79 (68.1)	108 (93.1)^a^
Basilic vein	9	6 (66.7)	9 (100.0)^a^^,^^b^
Axillary vein	51	29 (56.9)	49 (96.1)^a^
Superficial temporal vein	51	34 (66.7)	37 (72.5)^b^
Cephalic vein	15	6 (40.0)	14 (93.3)^a^^,^^b^
Great saphenous vein	2	0 (0.0)	2 (100.0)^a^^,^^b^
Fisher's exact test		8.68	16.48
*P*		0.1	0.003

^a,b,c^ Respectively represent different subsets of punctured blood vessel categories, there is no statistical difference between punctured blood vessels with the same letter, and *P* > 0.05.

## 4 Discussion

### 4.1 The total incidence of catheter-related complications is relatively low when mid-line catheters are inserted into the axillary vein and median cubital vein of neonates

Several studies have suggested that the use of MCs in this population can enhance indwelling time and decrease the occurrence of vascular access-related complications, thereby presenting a viable option for neonatal care (9). According to the INS guidelines, the preferred veins for MC insertion are primarily the median cubital, basilic, and cephalic veins in the upper-arm elbow region, with the basilic vein being the first choice (6). In cases of special circumstances such as pain, infection, vascular injury, or planned surgical procedures involving the puncture vein, an alternative vein should be selected (12). Evidence-based clinical practice guidelines for pediatric intravenous infusion therapy further indicate that the principal puncture vessels in neonates include not only the median cubital, basilic, and cephalic veins but also the superficial temporal, axillary, great saphenous veins, as well as the dorsal veins of the foot and hand (13). However, there is still a lack of research on the preferred veins for medium—length catheters in neonates.

The results of this study showed that there were significant differences in the incidence of complications when MC was inserted through different puncture vessels. The incidence of complications in the axillary vein and median cubital vein was lower than that in other puncture veins. This result suggests that the axillary vein and median cubital vein have the fewest complications during MC indwelling, which is consistent with the study by Chen et al. (14). The possible reason is that the median cubital vein and axillary vein in neonates are easier to expose and fix than the basilic vein. Moreover, the median cubital vein mostly drains into the axillary vein. The axillary vein is located in the armpit and is a branch of the subclavian vein. Its anatomical position is relatively fixed, with a thick and straight lumen and is not easy to slide. The puncture success rate is high, and the blood flow is good, which can quickly reduce the osmotic pressure of the liquid, reduce the irritation of hypertonic drugs to the blood vessels, maintain the integrity of the blood vessels, and thus reduce the occurrence of complications such as catheter occlusion, phlebitis, extravasation, and infection (15).

In addition, the results of this study also showed that the incidence of complications in the basilic vein was lower than that in the cephalic vein, superficial temporal vein, and great saphenous vein, and the difference was statistically significant when compared with the great saphenous vein. This suggests that when the median cubital vein and axillary vein cannot be selected for puncture, the basilic vein is also a relatively better choice. The possible reason is that compared with the basilic vein, the cephalic vein has an angle when draining into the axillary vein or subclavian vein and has more venous valves, resulting in unsmooth catheter insertion or repeated catheter insertions (16), which easily causes damage to the blood vessel wall and increases the risk of complications. Moreover, the cephalic vein is characterized by being thick at the front and thin at the back, with a relatively large catheter/vein ratio, which easily damages the venous wall during catheterization and increases the risk of phlebitis (17). Some studies have also shown that compared with the median cubital or basilic vein, inserting a medium—length MC through the cephalic vein is associated with a higher incidence of complications (14).

When puncturing through the superficial temporal vein, due to the abundant hair on the head, it is difficult to fix the catheter after placement, and the catheter is easy to fall off. In addition, the frequent movement of the child's head and neck causes friction between the catheter and the venous intima, increasing the risk of mechanical phlebitis and fluid leakage. Although previous literature has not highlighted the high risk of the great saphenous vein, our study identifies a relatively high incidence of complications in this vein. This may be attributed to the slower blood flow velocity in the lower-limb veins, which increases the risk of complications such as infection and thrombosis. In this study, a medium-length catheter measuring 10 cm was utilized. When inserted through the great saphenous vein, the catheter tip was predominantly positioned in the leg veins below the groin, where the lumen is relatively narrow and the blood flow velocity is reduced, thereby facilitating the occurrence of complications. Furthermore, the great saphenous vein serves as a critical alternative when upper limb vein catheterization is not feasible. Although only two patients were included in the great saphenous vein group, the 100% complication rate observed, in contrast to other catheterization sites, indicates significant risks for patients in extreme cases when this route is necessitated. Consequently, this group has been retained in the study for further analysis.

### 4.2 Neonates have a longer indwelling time of mid-line catheters when the catheters are inserted through the axillary vein and median cubital vein

Although the current guidelines for nursing practice in intravenous therapy suggest that the indwelling time of MC is 1–4 weeks (5), some studies have shown that the actual maximum indwelling time of the catheter can reach 131 days (16). Therefore, in clinical practice, whether the catheter needs to be removed is often judged based on its function during indwelling. This study found that there were statistically significant differences in the indwelling time of catheters among different puncture veins. The indwelling time when puncturing through the median cubital vein and axillary vein was better than that of other puncture veins, and there were statistically significant differences when comparing the median cubital vein and axillary vein with the superficial temporal vein. This result indicates that the vascular characteristics of different puncture veins have a significant impact on the indwelling time of the catheter. Therefore, it suggests that when neonates need long—term intravenous infusion and nutritional support, which require long—term indwelling of catheters, clinical nurses should give priority to puncturing through the median cubital vein and axillary vein to reduce the pain and infection risk caused by frequent catheter replacement for the infants. The possible reason is that when the catheter is inserted through the median cubital vein and axillary vein, the catheter tip is mostly located between the second segment of the axillary vein and the brachiocephalic vein, with a blood flow velocity of 5–30 cm/s. The relatively fast blood flow can quickly dilute the drugs, reducing the irritation of drugs to the vascular intima, thereby reducing the incidence of chemical phlebitis and catheter—related thrombosis. The fast blood flow can not only quickly dilute the drugs but also reduce blood coagulation, thus reducing the incidence of complications such as catheter occlusion and fluid leakage (17). When the catheter is inserted through the superficial temporal vein, the catheter tip is mostly located in the internal jugular vein. Even though the blood flow velocity in the internal jugular vein is relatively fast, the frequent movement of the newborn's neck will cause friction between the catheter tip and the vascular wall (18), which in turn increases the probability of vascular intimal damage, and easily leads to catheter displacement and fluid leakage from the puncture vein. In addition, in this study, the indwelling time of the cephalic vein and great saphenous vein was shorter than that of other puncture veins. The possible reason is that 52.94% (9/17) of the catheters inserted through the great saphenous vein and cephalic vein had complications. Among the 9 infants with complications, 6 had unplanned catheter removal, resulting in a shorter indwelling time, with the shortest being only 4 days. We presented the results of platelet and albumin in different groups. Platelets contribute to blood clot formation and release mediators that establish and sustain local inflammatory responses (19). A decrease in albumin may indicate a decrease in the patient's immune function. Therefore, patients with low albumin have a relatively high incidence of catheter complications (20). However, the levels of platelet and albumin had no significant difference among different puncture sites.

### 4.3 When inserting medium-length catheters into neonates via the axillary vein and median cubital vein, the catheter advancement is smoother, while there is no difference in the one-time puncture success rate

In previous studies (21–23), researchers found that when inserting MC via the upper arm vein or basilic vein, the one-time puncture success rate was high and the catheter advancement was smoother. The results of this study showed that there was no statistically significant difference in the one-time puncture success rate among different puncture veins for neonatal MC. In terms of the catheter advancement rate, the one-time successful catheter advancement rate of the median cubital vein and axillary vein was better than that of other puncture veins*.* This result indicates that there is no difference in the one-time puncture success rate among various puncture veins. This result suggests that in order to improve the one-time puncture success rate, more attention should be paid to the improvement of the operator's skills and the assessment of the patient's overall physiological state, rather than overemphasizing the influence of the choice of puncture vein on the one-time puncture success rate. The reason why the catheter advancement through the median cubital vein and axillary vein is smoother than that through other sites may be that the median cubital vein and axillary vein are relatively straight, have a larger diameter, and fewer venous valves (24), which makes it easier for the catheter to pass through smoothly. This result helps to optimize the clinical operation process of intravenous puncture and catheterization. When intravenous catheterization is required, the median cubital vein and axillary vein should be considered first to improve the success rate of one-time successful catheter advancement and reduce the patient's pain and the occurrence of complications. Some studies have also reported the advantages of the median cubital vein and axillary vein in the puncture operation of peripherally inserted central catheters (25–27), which is consistent with the result of the relatively high one-time successful catheter advancement rate of the median cubital vein in this study.

## 5 Limitations

The sample in this study is exclusively derived from neonates in the NICU of our hospital, which introduces both selection and information biases, thereby limiting the generalizability of the findings. Additionally, the sample size for great saphenous vein puncture is limited to only two cases. Despite the observed complication rate of 100%, it is imperative to expand the sample size to further assess the risks associated with this site. Furthermore, whether different puncture sites are risk factors for complications should be analyzed using multiple regression analysis on the premise of expanding the sample size. In addition, the study did not account for the impact of body weight dynamics on complications, and potential confounding factors were not addressed. Future research should aim to provide a more robust foundation for determining the optimal puncture site for neonatal MC by engaging in multi-center collaborations, increasing the sample size for the great saphenous vein, and incorporating gestational age matching and biomarker analysis. This study is a single center retrospective study. Although the sample has a certain representativeness, future multicenter, prospective cohort studies are needed to further verify the association between puncture methods and complications, especially the differences in effectiveness under different medical resource configurations.

## 6 Conclusion

When selecting puncture veins for mid-line catheters in neonates, the axillary vein and median cubital vein offer distinct advantages concerning catheter indwelling time, catheter puncture and advancement, and complication rates. Nonetheless, it is imperative in clinical practice to holistically assess the specific conditions of neonates, including the nature of the illness and the feasibility of accessing the puncture veins, to determine the most suitable puncture vein. This comprehensive approach aims to enhance the safety and efficacy of mid-line catheter applications in neonatal care.

## Data Availability

The raw data supporting the conclusions of this article will be made available by the authors, without undue reservation.
